# Supplementary material for: Provision and delivery of survivorship care for adult patients with haematological malignancies: A scoping review protocol
Source: PLoS One. 2023 Mar 2;18(3):e0282458. doi: 10.1371/journal.pone.0282458 (PMC9980752; doi:10.1371/journal.pone.0282458)
Supplement: S1 Timeline — (DOCX) [file pone.0282458.s002.docx]

**Scoping Review – Proposed Timeline**

|  | 2022 | | | | | | | | 2023 | | | | | | | | | | |
| --- | --- | --- | --- | --- | --- | --- | --- | --- | --- | --- | --- | --- | --- | --- | --- | --- | --- | --- | --- |
| *Activity* | **May** | **Jun** | **Jul** | **Aug** | **Sep** | **Oct** | **Nov** | **Dec** | **Jan** | **Feb** | **Mar** | **Apr** | **May** | **Jun** | **Jul** | **Aug** | **Sep** | **Oct** | **Nov** |
| Develop protocol |  |  |  |  |  |  |  |  |  |  |  |  |  |  |  |  |  |  |  |
| Register protocol |  |  |  |  |  |  |  |  |  |  |  |  |  |  |  |  |  |  |  |
| Conduct searches |  |  |  |  |  |  |  |  |  |  |  |  |  |  |  |  |  |  |  |
| Protocol edits |  |  |  |  |  |  |  |  |  |  |  |  |  |  |  |  |  |  |  |
| Additional searches |  |  |  |  |  |  |  |  |  |  |  |  |  |  |  |  |  |  |  |
| Study selection |  |  |  |  |  |  |  |  |  |  |  |  |  |  |  |  |  |  |  |
| Submit protocol article |  |  |  |  |  |  |  |  |  |  |  |  |  |  |  |  |  |  |  |
| Data extraction |  |  |  |  |  |  |  |  |  |  |  |  |  |  |  |  |  |  |  |
| Summarise data |  |  |  |  |  |  |  |  |  |  |  |  |  |  |  |  |  |  |  |
| First  draft |  |  |  |  |  |  |  |  |  |  |  |  |  |  |  |  |  |  |  |
| Second draft |  |  |  |  |  |  |  |  |  |  |  |  |  |  |  |  |  |  |  |
| Present results |  |  |  |  |  |  |  |  |  |  |  |  |  |  |  |  |  |  |  |
| Final  draft |  |  |  |  |  |  |  |  |  |  |  |  |  |  |  |  |  |  |  |
| Submit results article |  |  |  |  |  |  |  |  |  |  |  |  |  |  |  |  |  |  |  |

Key: Proposed Completed In progress
