# Supplementary material for: Provision and delivery of survivorship care for adult patients with haematological malignancies: A scoping review protocol
Source: PLoS One. 2023 Mar 2;18(3):e0282458. doi: 10.1371/journal.pone.0282458 (PMC9980752; doi:10.1371/journal.pone.0282458)
Supplement: S1 File — (DOCX) [file pone.0282458.s003.docx]

**Scoping Review – Example MEDLINE Search Strategy**

*Ovid MEDLINE(R) ALL 1946 to August 16, 2022*

1. exp leukemia, hairy cell/ or exp leukemia, lymphoid/ or exp leukemia, mast-cell/ or exp leukemia, myeloid/ or exp leukemia, plasma cell/ or exp leukemia, radiation-induced/

2. exp Lymphoma/

3. exp Hematologic Neoplasms/

4. exp Myelodysplastic Syndromes/

5. exp Myelodysplastic-Myeloproliferative Diseases/

6. exp leukemia, erythroblastic, acute/ or exp leukemia, myelogenous, chronic, bcr-abl positive/ or exp leukemia, neutrophilic, chronic/ or exp polycythemia vera/ or exp primary myelofibrosis/ or exp thrombocythemia, essential/

7. (h?ematolog* cancer* or h?ematolog* malignanc* or h?ematolog* oncolog* or h?emato-oncolog* or h?ematolog* neoplasm* or blood cancer* or non-solid tum?or* or leuk?emia* or lymphoma* or myeloma* or Hodgkin* disease or myeloproliferative neoplasm* or myelodysplasia or myelodysplastic syndrome*).ab,kf,kw,ti.

8. 1 or 2 or 3 or 4 or 5 or 6 or 7

9. exp Cancer Survivors/

10. exp Survivorship/

11. (survivor* or "living ADJ4 cancer").ti,ab,kw,kf.

12. 9 or 10 or 11

13. exp Follow-Up Studies/

14. exp Patient-Centered Care/

15. exp Neoplasms, Second Primary/

16. exp Watchful Waiting

17. ("after ADJ2 treatment*" or "following ADJ2 treatment" or "post ADJ2 treatment" or post-treatment or "end ADJ3 treatment" or "complet* ADJ2 treatment" or "follow up" or follow-up or followup or "late ADJ2 effect*" or "adverse ADJ2 effect*" or "treatment ADJ2 effect" or "chronic AJD2 symptom*" or "chronic ADJ2 effect*" or recovery or rehab* or wellbeing or well-being or "personali#ed care" or stratif* or "patient cent?red" or patient-cent?red or "model* ADJ4 care" or "care plan*" or "treatment ADJ2 summar*" or "long ADJ2 effect*" or consequence* or long-standing or recurrence* or secondar* or "new primar*" or "new malignanc*" or "new cancer*" or "subsequent primar*" or "subsequent malignanc*" or "subsequent cancer*" or surveillance or "care coordination" or "care co-ordination" or aftercare or "shared care").ti,ab,kw,kf.

18. 13 or 14 or 15 or 16 or 17

19. 8 and 12 and 18

20. (exp adolescent/ or exp child/ or exp infant/ or exp pediatrics/ or exp young adult/) not exp Adult/

21. 19 not 20

22. (animals not humans).sh.

23. 21 not 22

24. limit 23 to yr="2007 -Current"
